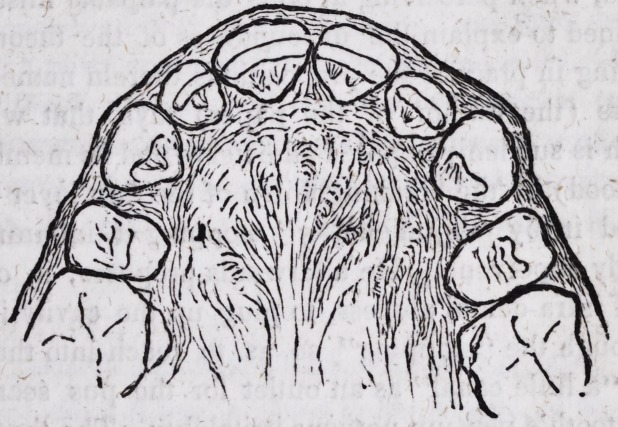# Irregularity of the Superior Denture

**Published:** 1843-09

**Authors:** 


### ARTICLE VII.

*Irregularity of the Superior Denture.* Treated by the JUNIOR EDITOR.

THE patient was a healthy, well grown boy of 15 years. The position of his teeth, at his first visit to me, will best be understood by referring to this engraving.

As no notes were taken during the treatment, I am unable to give the time required for each step of the cure. I saw the patient every few days, and made the applications herein described, as changes in the positions of the teeth indicated their propriety. On the 7th of September, I extracted both the first superior bicuspides, and directed the patient to return as soon as the inflammation consequent upon this operation should have slightly subsided. Having met with an accident, he did not return for several weeks. At the next visit, I passed an elastic thread, (made of sewing thread and gum-elastic,) around behind the first molar and before the cuspidatus of one side; the two ends of the thread were then tied tightly, and the corresponding teeth of the other side were treated in like manner. These threads were several times renewed as shorter ones became necessary. As both sides were treated alike it will be sufficient to particularize the treatment of but one of them. Having drawn the cuspidatus about one line back, thread was now passed between the bicuspis and first molar; then the end towards the cheek was passed between the first and second molares; this gave both ends toward the palate; then both ends were brought forward and tied tightly before the cuspidatus, thus changing the direction of the force by making the bicuspis serve as a pulley. But this pulley would serve as such but a short time, becoming, itself, loose in a few days. I then let the threads of both sides remain to keep the cuspidati from returning while the parts adjacent to them were allowed to rest a few days and recover from some slight soreness, and directed the treatment to the incisores.

A slip of gum-elastic was placed between each lateral incisor and its adjoining central, which separated the teeth at these places about half a line. A stick of hickory pivoting was then split in two, and cut of the right length to reach from the depression in the back of one lateral incisor to that in the other. This was cut away toward each end so as to form a spring, thickest in the middle, where a non-elastic thread was tied fast. This spring was placed against the backs of the lateral incisores and held there by the patient pressing forcibly against its middle, while both ends of the thread were brought forward between the central incisores and tied tightly over a thin stick two lines long of the same wood, laid across the space between the centrals. This carried the lateral incisores forward and outward, while it slightly obtunded the angle formed by the centrals. This last effect was sufficiently produced afterward by applying a like spring, in like manner, except that its ends rested upon the backs of the central incisores, at those parts nearest to the laterals.

A like spring of hickory was now applied to bring the cuspidatus of each side toward the palate, by having one end rest upon the palatine side of the first molar, and the other upon the back of the lateral incisor. This brought the latter to its place, but the cuspidatus was still a little too prominent. One end of a non-elastic thread was now passed between one lateral incisor and its adjoining cuspidatus—through to the front between this lateral and its adjoining central—across the front of both centrals—through between the other lateral and its adjoining central—through to the front next the other cuspidatus, and there tied across the front of all the incisores, moderately tight, to the other end. This kept the incisores from change of position while the elastic thread was again applied to the cuspidati, using the bicuspides as pulleys as before. This brought the cuspidati into place, and the teeth were then regular. Non-elastic threads were now applied to the cuspidati, in the same manner as those they superseded, and these and the one upon the incisores were kept upon the teeth two or three weeks and then removed. After a week or two one of the lateral incisores was found to be a little within its proper place. A tooth immediately under it, in the inferior denture, being found to be also within its proper arch, was brought to the front by the application of means similar to those detailed; and being held there a few days brought the upper one to its proper position by pressure upon its back. After a few days wearing the thread upon the under teeth, it was removed and the cure was completed, as represented in this engraving:—the treatment having lasted, (including lost time,) some four or five months.

It will be seen from this account that no metallic bars, frames, blocks, hooks or springs, in fact, no metal or anything of like rigidity was used. The patient attended to his studies and other duties as he would have done without the treatment; suffering little if any pain, and but slight inconvenience. Probably much less time would have been required for the operation, but for the accident which delayed it at the commencement. M.

*Washington, Aug. 10th, 1843.*

## Figures and Tables

**Figure f1:**
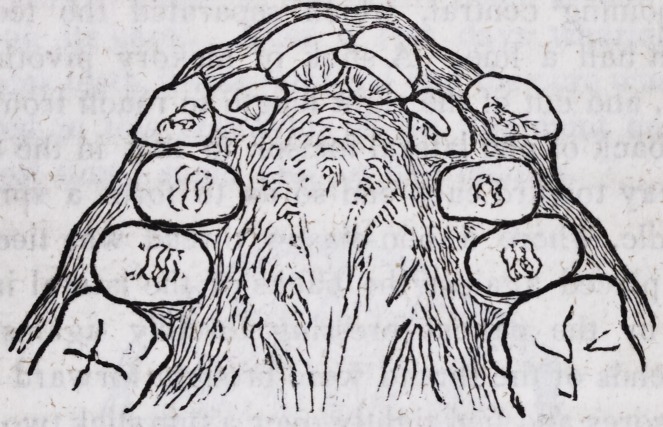


**Figure f2:**